# Biomechanical evaluation of custom-made short implants with wing retention applied in severe atrophic maxillary posterior region restoration: A three-dimensional finite element analysis

**DOI:** 10.3389/fbioe.2023.1137779

**Published:** 2023-02-09

**Authors:** Zhen Yang, Jingran Zhang, Zexian Xu, Xiaoqiang Liu, Jianjun Yang, Jianguo Tan

**Affiliations:** ^1^ Department of Prosthodontics, Peking University School and Hospital of Stomatology, Beijing, China; ^2^ Peking University School and Hospital of Stomatology & National Center of Stomatology & National Clinical Research Center for Oral Diseases & National Engineering Laboratory for Digital and Material Technology of Stomatology & Beijing Key Laboratory of Digital Stomatology & Research Center of Engineering and Technology for Computerized Dentistry Ministry of Health & NMPA Key Laboratory for Dental Materials, Beijing, China; ^3^ Department of Periodontology, Peking University School and Hospital of Stomatology, Beijing, China; ^4^ Department of Oral and Maxillofacial Surgery, the Affiliated Hospital of Qingdao University, Qingdao, China; ^5^ School of Stomatology of Qingdao University, Qingdao, China

**Keywords:** atrophy, finite elements, stress distribution, dental implant, custom-made

## Abstract

Severe bone atrophy in the maxillary posterior region poses a big challenge to implant restoration. Digitally designed and customized short implants with wing retention provide a safer and minimally invasive implant restoration scheme in such circumstances. Small titanium wings are integrated with the short implant supporting the prosthesis. Using digital designing and processing technology, the wings fixed by titanium screws can be flexibly designed, providing the main fixation. The design of the wings will influence the stress distribution and implant stability. This study analyzes the position, structure, and spread area of the wings fixture scientifically by means of three-dimensional finite element analysis. The design of the wings is set to linear, triangular, and planar styles. Under the simulated vertical and oblique occlusal forces, the implant displacement and stress between the implant and the bone surface are analyzed at different bone heights of 1 mm, 2 mm, and 3 mm. The finite element results show that the planar form can better disperse the stress. By adjusting the cusp slope to reduce the influence of lateral force, short implants with planar wing fixtures can be used safely even if the residual bone height is only 1 mm. The results of the study provide a scientific basis for the clinical application of this new customized implant.

## 1 Introduction

Alveolar bone resorption occurs due to local inflammation and a long-time lack of physiological stimulation after tooth loss. Pneumatization of the maxillary sinus is more likely to lead to a severe reduction of residual bone height (RBH), which brings a big challenge to implant restoration in the maxillary posterior region (Bitinas and Bardijevskyt, 2021). According to the 6th ITI Consensus, short implants of a diameter ≤6 mm can be chosen as a valid treatment option in atrophic ridge cases. However, studies have revealed that they have a higher variability and lower predictability in survival rates (85%–100%) than standard ones (95%–100%) (Jung et al., 2018). Sufficient bone tissue is considered a critical condition for implant anchorage. Therefore, transalveolar sinus floor elevation (tSFE) with simultaneous implant placement is proposed in situations with RBH above 4 mm (Pjetursson and Lang, 2014; Qian et al., 2020). However, it has been found that the RBH of some patients is below 4 mm, and sometimes even only 1–2 mm. Weaker maxillary sinus floor bones increase the risk of maxillary sinus membrane perforation in tSFE treatment, so lateral sinus floor elevation with delayed implant placement is recommended. However, this technique would prolong the recovery time and cause more suffering for patients (Gonzalez et al., 2014; Testori et al., 2019). Moreover, when the RBH is below 1.5 mm, only cortical bone is left, and effective graft regeneration may not be possible (Taschieri et al., 2015).

Zygomatic implants reported by Bedrossian are developed to use when the RBH in the maxillary posterior area is extremely insufficient. This implant is about 30–52.5 mm in length and should be placed via the sinus cavities and anchored in the zygoma for stability. A few cases have shown favorable results for this technique, but further long-term clinical observation is still lacking (Varghese et al., 2021). In addition, the technique is more invasive and complex, and is often associated with serious complications such as infection, bleeding, and nerve damage. Therefore, it is not widely used in clinics. For patients with severe bone deficiency in the maxillary posterior region, especially those with RBH of less than 3 mm, there is no good clinical treatment at present. Subperiosteal implants have been re-proposed following the development of modern digital dentistry (Gellrich et al., 2017). The subperiosteal implants gain stability through the use of large-area spread titanium plate fixation. Although the plate can be individually designed to ensure fitness, poor blood flow of soft tissue still can be caused by extensive flap surgery. Nevertheless, the use of subperiosteal implants has facilitated the conception and development of innovative bone anchorage systems for oral restorations.

We have developed a new type of implant named Yang’s Implant (Xu et al., 2022). This implant, as described above, is composed of a short implant and retaining wings. But unlike the subperiosteal implant, Yang’s implant has an implanted part and abutment structure, and the platform switching structure is maintained to ensure soft tissue closure formation around the implant after implantation. The wing retention is fixed by titanium screws, which can be flexibly designed and provide the main fixation. In the circumstance of RBH being less than 4 mm, the stability and stress condition of Yang’s implant is still unclear. However, three-dimensional finite element analysis (3D-FEA) models have been established, which are useful to guide the design and innovation of Yang’s implant to better realize the clinical applications.

## 2 Materials and methods

### 2.1 Sinus geometric modeling

Initial data was obtained from patients’ CBCT. The thresholding operation was performed to extract the relevant structural information of the maxilla to reconstruct the point cloud data model, then geometric modeling and a three-dimensional (3D) finite element model of the maxillary sinus were carried out with the software Mimics 24.0 (Materialises, Leuven, Belgium). After that, the local finite element model was refined with the software Hypermesh 2017 (Altair, Troy, USA) to generate an editable maxillary sinus model with RBH of less than 4 mm. The height of RBH was set up to 1 mm, 2 mm, and 3 mm, respectively.

### 2.2 Yang’s implant geometric modeling

**FIGURE 1:**
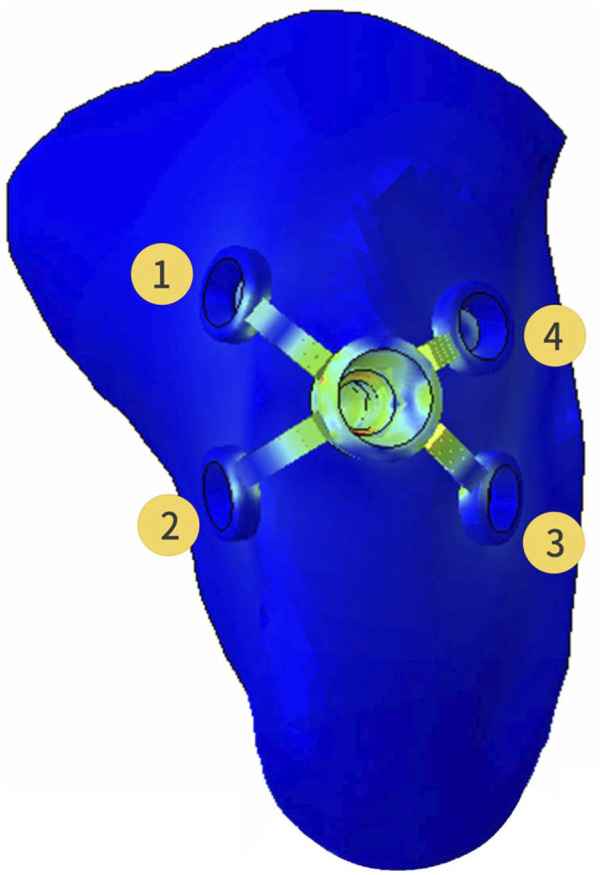
Three-dimensional finite element model of the partial maxilla and Yang’s implant; Representative numbers of different direction wings.

Registered CBCT data with model scanning data was used to obtain a virtual 3D bone reconstruction model of the patient. The position and shape of Yang’s implant was designed by 3Shape dental system (3Shape, Copenhagen, Denmark) as described previously (Xu et al., 2022), and the associated file was saved in standard tessellation language (STL) format. The STL format file was imported into Hypermesh software to form the Yang’s implant network model. The position of the wings responsible for retention of the implant could be moved and adjusted in this model. In this study, the implant restoration model of a left upper first molar was established. The wings were distributed in different directions on the buccal or lingual side, and they were represented with numbers, as shown in Figure 1 *.* Named wing numbers of 13 as linear style (LS), 134 as triangular style (TS), and 1234 as planar style (PS), and the names were used for subsequent analysis.

### 2.3 Model assembly and material properties

**FIGURE 2:**
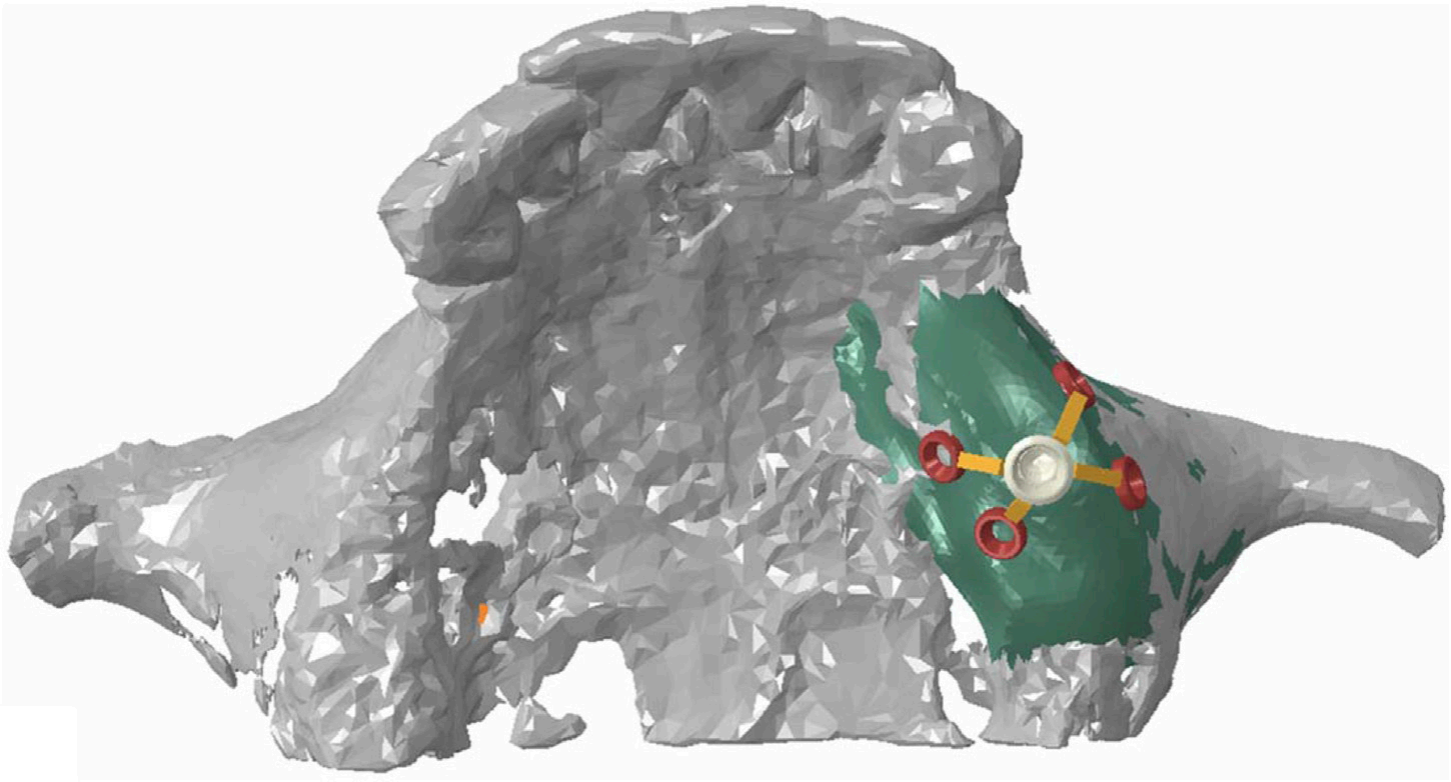
Assembly diagram of maxillary model and Yang’s implant model.

The refined mesh model of the maxillary sinus was generated into a local geometry model, and the generated local geometry model was combined with the model of the implant through Boolean operations (Figure 2).

The material properties of different kinds of tissues and implants in the model were set to be homogeneous, isotropic, and linear elastic. The Young’s modulus and Poisson’s ratio of materials shown in Table 1 were taken from the previous study (Yan et al., 2015).

**TABLE 1:** Material properties.

Material	Young’s modulus (MPa)	Poisson’s ratio
**Titanium implant**	103,400	0.35
**Cortical bone**	13,700	0.3
**Cancellous bone (D3)**	1370	0.3
**Sinus membrane**	58	0.45

### 2.4 Interface conditions

To obtain the initial stability of Yang’s implant, the interface between the implant and bone was assumed as a friction interface. It was modeled using non-linear frictional contact elements that allow for tiny displacements between the implant and the bone. The friction coefficient between the implant and bone/callus was set to 0.2. After 3 months, it was determined that osseointegration was formed. The friction coefficient was adjusted to infinity and tested the stress distribution of Yang’s implants on the bone under the situation of different directions of force.

### 2.5 Loading and boundary conditions

Parts of the maxillary sinus model that interfered with the implant were removed. The maxillary sinus mesh in the hole-edge area was reconstructed and optimized to ensure the quality of the calculation mesh. The maxillary sinus and implant model was assigned to the unit attribute, the unit type was set to higher-order tetrahedron C3D10M.

**FIGURE 3:**
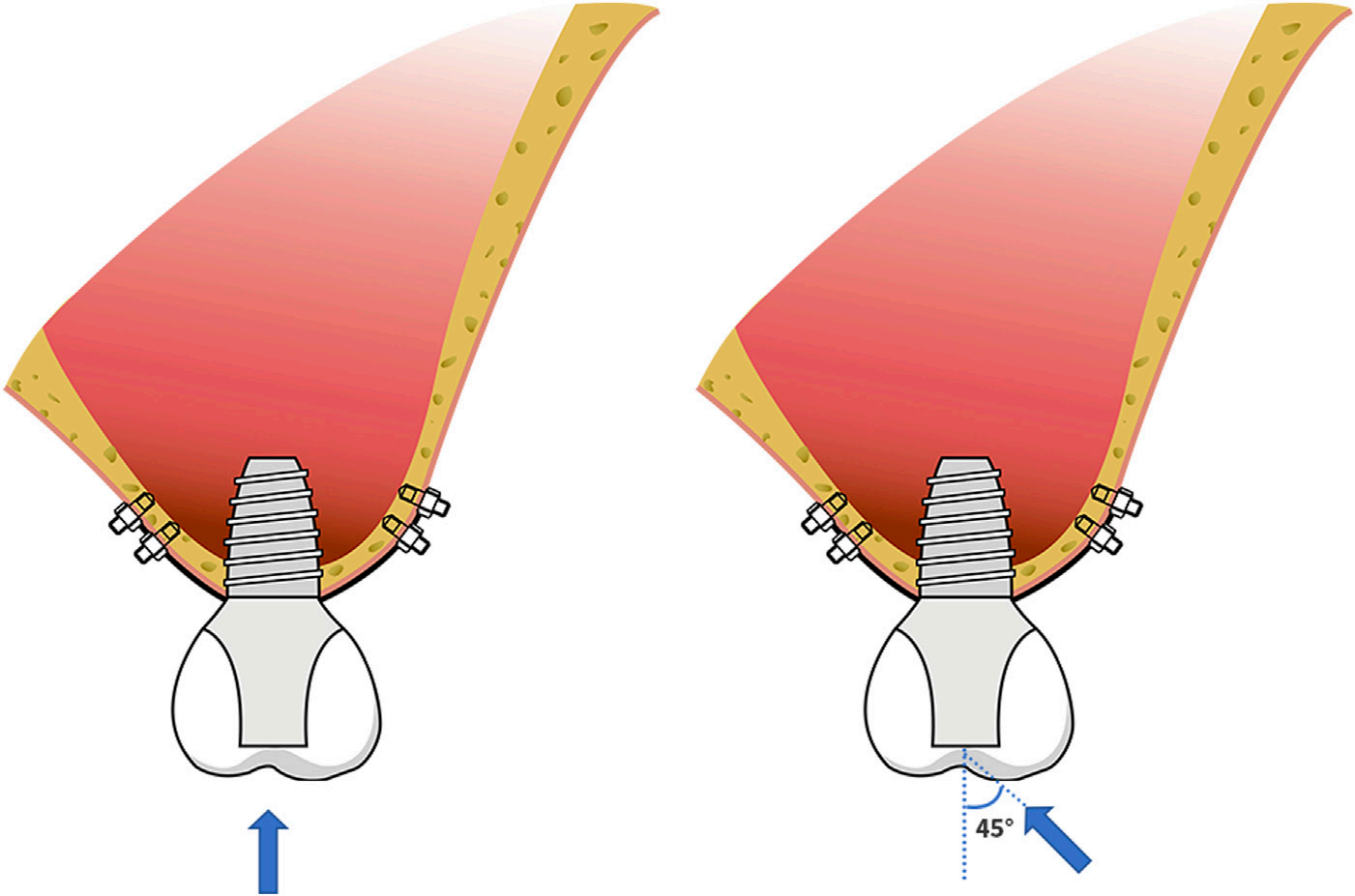
Schematic diagram of force loading; the force of 150 N loaded in a vertical direction on the top of the crown (0°) and at an angle of 45° (45°) to the long axis of the crown.

The average occlusal force of 150 N was loaded in a vertical direction on the top of the crown (0°) and at an angle of 45° (45°) to the long axis of the crown (Figure 3). Abaqus 2018 software (Dassault Systèmes, Paris, France) was used for calculation, and the results were outputted after post-processing. Different RBH heights were set and measured the von Mises stress at the implant-bone interface of differently designed implants. To assess the distribution of stresses, von Mises stresses were visualized with stress contour plots. Biomechanical effects were also analyzed by comparing the maximum displacement of the implants.

When the RBH was only 1mm, a force of 150 N was applied at angles of 20° and 30° to the long axis of the crown, respectively. Calculations were made with the same method. The von Mises stress at the implant-bone interface in the LS, TS, and PS groups was recorded and compared with the stress at 0° and 45°.

## 3 Results

### 3.1 Implant displacement

**FIGURE 4:**
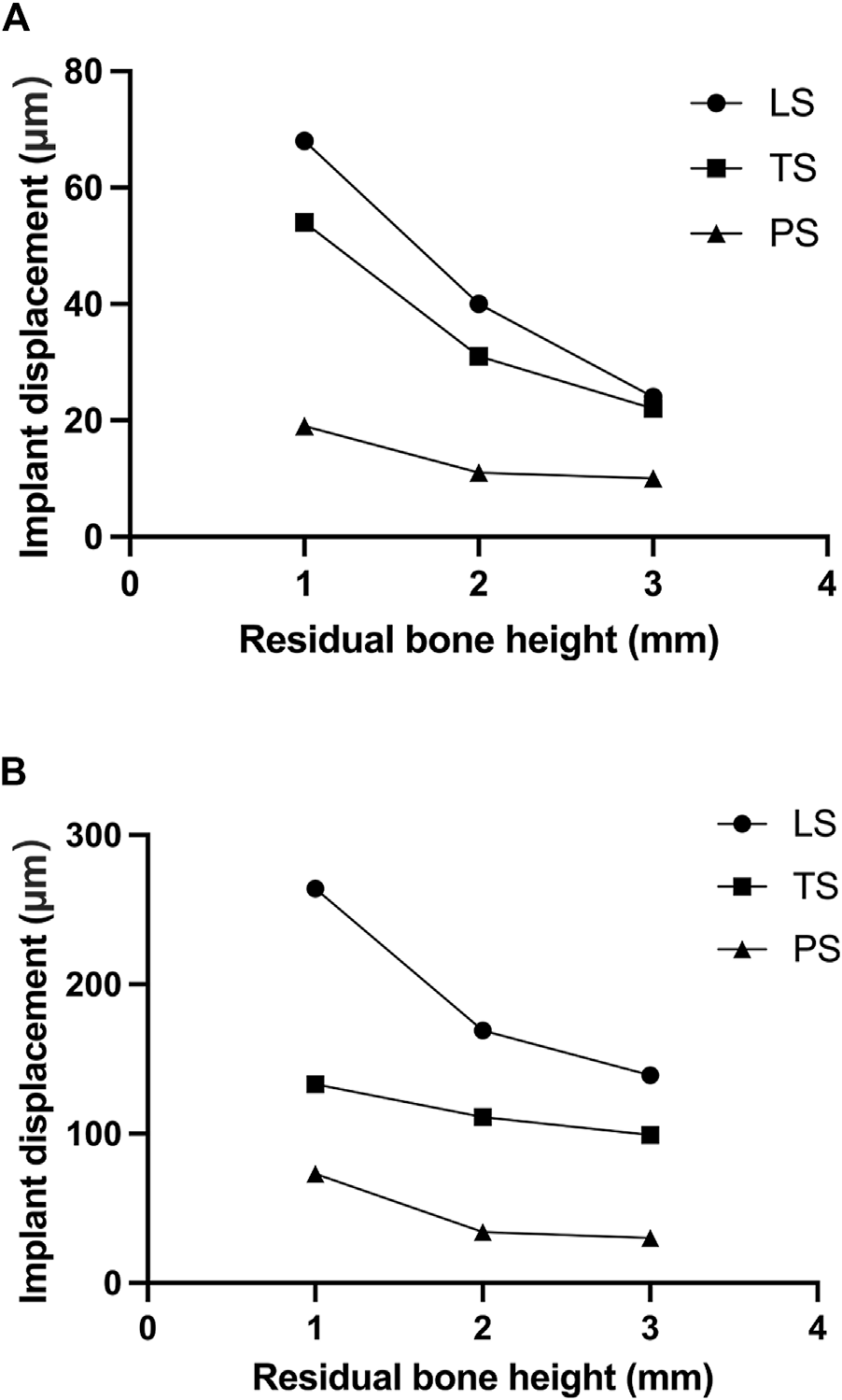
Implant displacement changes of different residual bone heights and different wing designs under forces in different directions; **(A)** 0°; **(B)** 45°.

The change of RBH, the direction of force, and the design of implant wing retention all affected the displacement of the implant. With the decrease of RBH, the displacement of the implant increased. The PS group could control the implant displacement by about 11.5 μm when the RBH remained 2 or 3 mm and the force was given perpendicularly. Under such conditions, the implant displacements of groups LS and TS were higher, at about 22.1 μm at 3 mm and 32.1 μm–40.3 μm at RBH 2 mm. In the case where the RBH was reduced to 1 mm, implant displacements of the LS and TS groups were obviously higher, exceeding 50 μm. However, the PS group would better maintain the displacement of the implant at 19.5 μm (Figure 4A). Compared to the force direction of 0°, a 45° force would shapely increase the displacement of the implant. Nevertheless, the planar retention style better maintained the implant stability compared to the other two groups. When RBH was reduced from 3 mm to 1 mm, the maximum displacement of the implant could still be controlled below 100 μm (Figure 4B).

### 3.2 Stress distribution at the implant-bone interface

**FIGURE 5:**
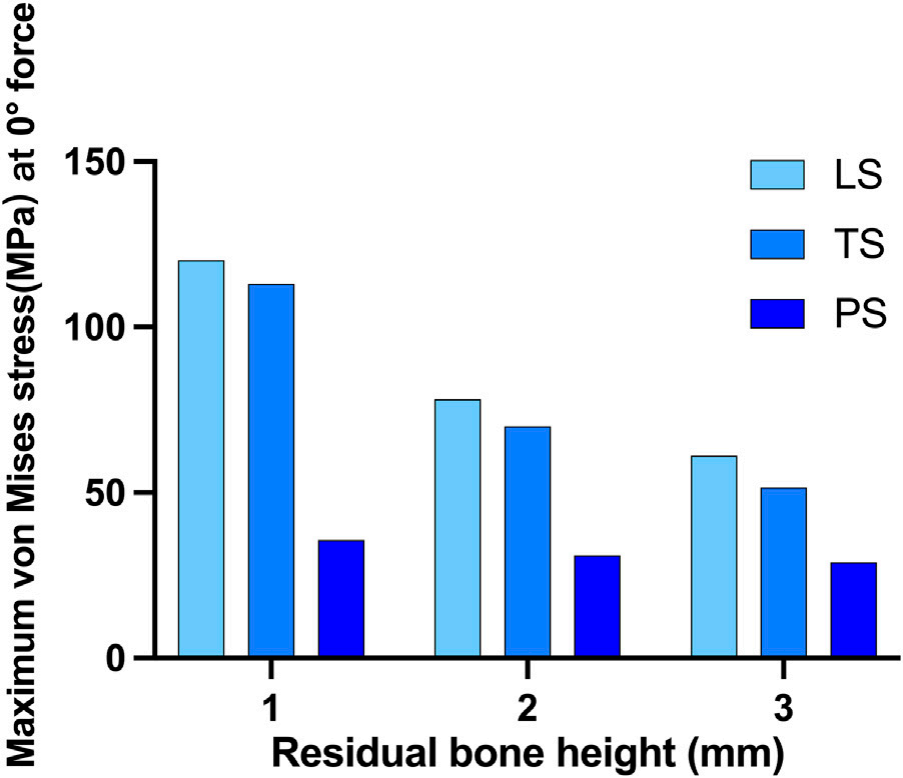
Maximum von Mises stress on crestal cortical bone of different residual bone heights and different wing designs at 0° force.

The highest stress on the bone tended to increase as the height of the alveolar ridge decreased. Stress was gathered on the cortical bone around the neck of the implant and the retention wing. A force of 150N was applied perpendicularly to the direction of the crown. When the RBH was 3 mm, the maximum von Mises stress of the LS and TS groups was above 50 MPa, and the LS group was much higher than the TS group, at about 65.1 MPa. Compared to these two groups, the PS group was much lower, at about only 30.2 MPa. The maximum von Mises stress on crestal cortical bone slowly increased when RBH was decreased to 2 mm. The LS and TS groups could maintain the stress around 65.4–70.8 MPa, while the PS group kept the stress still below 40 MPa. However, the stress in the LS and TS groups increased significantly when the RBH reached 1 mm; compared to RBH 2 mm, the stress nearly doubled, soaring to 120.3 MPa. In these circumstances, the wing design of the planar style had outstanding advantages. The stress in this group could be controlled stably below 40 MPa (Figure 5).

**FIGURE 6:**
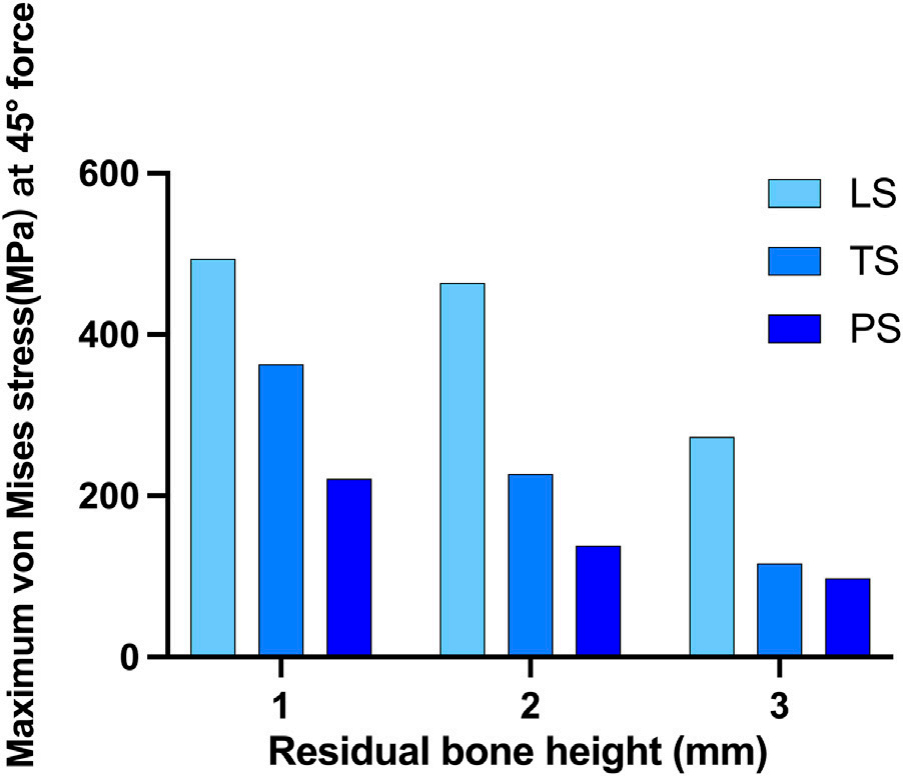
Maximum von Mises stress on crestal cortical bone of different residual bone heights and different wing designs at 45° force.

When the force was applied at 45° oblique to the direction of the crown, the maximum von Mises stress on the cortical bone obviously increased. In this situation, much of the force was concentrated around the implant neck. The linear style wing design was not suitable for resisting the 45° force even when the RBH was 3 mm, and the maximum stress far exceeded 200 MPa. Compared with the LS group, group TS reduced the stress to about 116.2MPa, and the lowest stress was shown in group PS, at about 91.9 MPa. However, even using the planar design form, when RBH remained only 1 mm or 2mm, the pressure on the cortical bone would increase at a faster rate. The stress reached 158.1 MPa at RBH 2 mm and 218.5 MPa at RBH 1 mm. Additionally, maximum stress also increased in group TS and group LS when RBH was less than 3 mm, to about 225.1–355.7MPa and 434.2–482.7 MPa, respectively (Figure 6).

**FIGURE 7:**
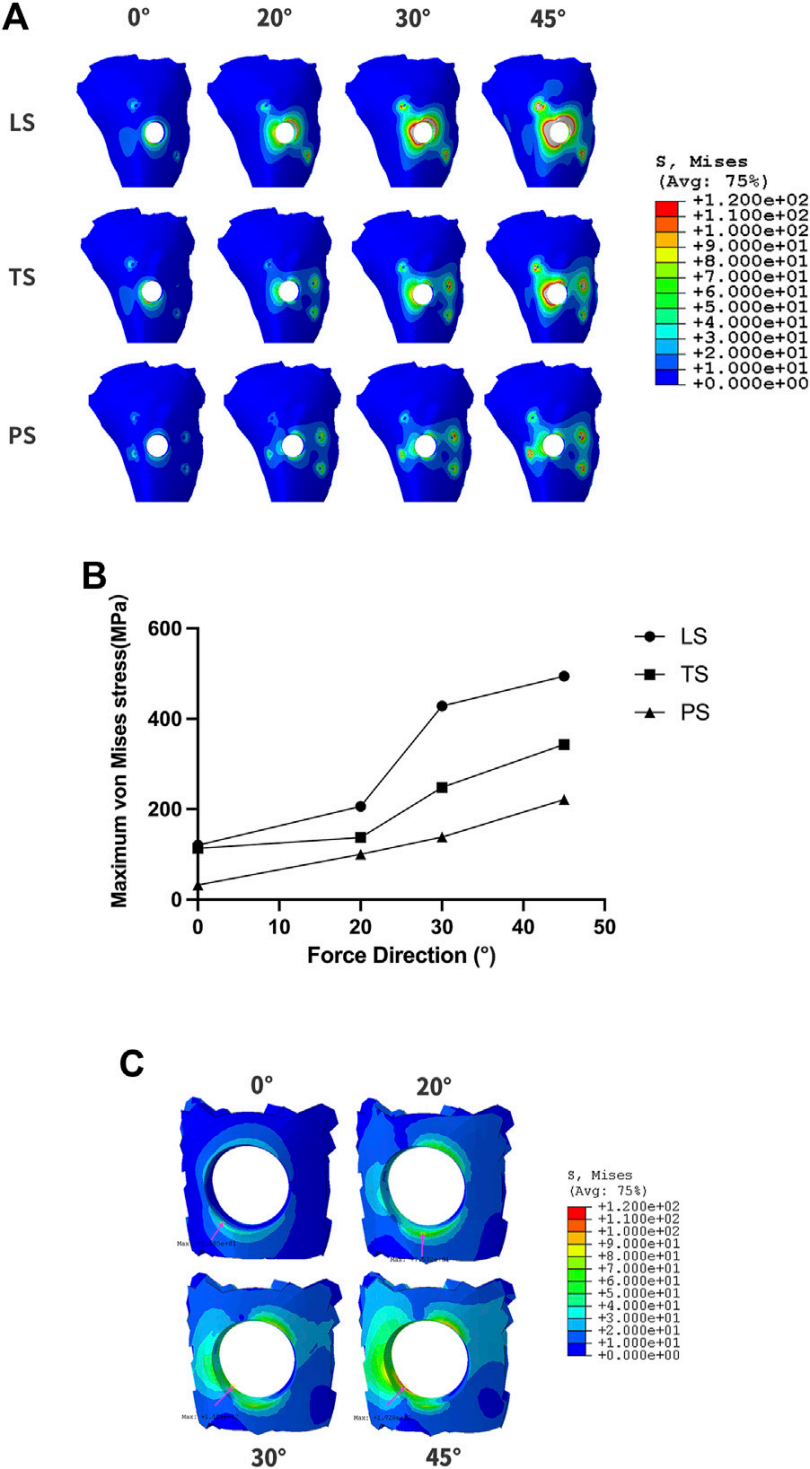
The changes of maximum von Mises stress according to different force directions and wing designs at RBH 1 mm. **(A)**. The diagram of maximum von Mises stress distribution; **(B)**. Value changes of maximum von Mises stress; **(C)**. The stress distribution at the hole-edge of the PS group.

When RBH was only 1mm, more stress distribution showed regardless of the wings’ forms. Clinically, the impact of adverse lateral force on implant restorations could be reduced by changing the cusp slope of the crown, and adjusting the direction of the force to represent the inclination adjustment of the cusp. We applied a 150N force oblique to the crown at an angle of 20° and 30°, respectively, to test the stress distribution. As the force moved, stress gathered around the implant neck gradually shifted from the center to the opposite side of the force (Figure 7A). When the force was set to 20°, the maximum stress in group LS was about 200 MPa, and the stress in group TS could be controlled at 157.1 MPa. When the force was changed to 30°, the maximum stress in the LS group increased obviously to 410.7 MPa, and also increased in the TS group, reaching 228 MPa. For the PS group, when the force was given at 45°, the maximum stress exceeded 200 MPa. When the force direction was changed to 20° or 30°, the maximum stress could be well controlled below 150 MPa (Figure 7B). In group PS, when the vertical force or 20° force was given, the maximum stress concentration was at the edge of the hole on the outside surface of the cortical bone. When 30° and 45° forces were applied, the greatest stress concentration existed at the medial cortical bone of the hole margin (Figure 7C).

## 4 Discussion

The concept of submucosal implants was first proposed in the 1940s. Initially, this implant was placed under the periosteum, anchored directly to the bone surface by means of a large-area spread plate *via* screw fixation (Silvestri and Carlotti, 1995). It solved the restoration problem of patients with severe bone atrophy to some extent. Nevertheless, the use of submucosal implants decreased in the late 1970s. One of the important reasons for the rapid decline of subperiosteal implants was that the fixed plate and post-column were integrally cast, so tiny mucosa inflammation could spread rapidly and lead to failure (Schou et al., 2000; Nemtoi et al., 2022). With the development of digital dentistry, subperiosteal implants have been re-proposed (Gellrich et al., 2017; Nemtoi et al., 2022). CAD/CAM techniques can make the retained titanium plate closely fit the bone surface in order to obtain satisfactory retention. However, the problem of one-piece manufacturing is not solved. The wing retention of the Yang’s implant we developed borrows the idea of the retention plate from submucosal implants. But our implant has an implanted part, and the implant and the abutment are two sections, between which a platform switching structure is used. The platform switching connection is proven to have a better soft tissue seal around the implant, thus effectively preventing infection and marginal bone resorption. Meanwhile, Yang’s implant can be customized with CAD/CAM techniques and precisely machined with a seven-axis lathe, so that the wings can fit tightly to the bone surface and form good retention.

3D-FEA has been widely used in dental research. It can be an excellent method for modeling complex structures and analyzing their mechanical properties (Trivedi, 2014). With this technique, it is possible to simulate complex structures on a microscopic scale to observe further stress distribution that is clinically impossible to observe (Turker et al., 2021). Yang’s implant provides an effective method for implant restorations of patients with RBH less than 4 mm. In this circumstance, reliance on osseointegration between the implant component and the residual bone will not be sufficient for effective primary stability. The wing retained by titanium screws around the implant can provide critical retention. Larger wing spreading areas bring better support, but a larger flap elevation area is also required during the operation, which does harm to the blood supply of the soft tissue. Additionally, the direction of the wing also affects the stress distribution, thus influencing the stability of the implant. In this study, 3D-FEA was established to analyze the stress distribution of Yang’s implant with different designs and numbers of wings at different RBHs. During the process of implant surgery, the gingival flap is first performed, then the implant is placed. After the surgery, the gingiva is sutured. The most important function of the healed gingiva is to form a good soft tissue seal around the implant neck to prevent complications such as infection. It has no effect on the osseointegration stability of Yang’s implant, so the gingival factor was not analyzed in the model. The result of the study is significant for further clinical design and application.

Micromotion is defined as a phenomenon that occurs at the interface of two components leading to the displacement of one component relative to the second one (Winter et al., 2013). Large micromotion at the bone-implant interface is harmful. Over 150 μm micromotion will induce the formation of fibrous connective tissue, thus interfering with implant osseointegration (Brunski, 1993; Szmukler-Moncler et al., 1998; Barnes et al., 2019). The wing retention structures of Yang’s implants mainly account for the primary implant displacement. From the results of the study, a linear wing form has a poor ability to stabilize the initial displacement of the implant, especially under an oblique force. The triangular design is more conducive to the spread and dispersion of force. The planar style could distribute the occlusal force over surrounding cortical bone, effectively controlling the distribution of the implant and maintaining initial stability. When lateral force is applied, the implant displacement could still be guaranteed to be less than 100 μm even if the RBH is 1 mm. From the perspective of displacement control, the planar design form could better meet clinical application requirements.

According to Wolff’s law of bone transformation, the bone’s response to absorption or healing is directly related to stress in the bone (Frost, 1994). Excessive distribution of stress concentration is one of the important factors involved in time-dependent marginal bone loss, with inevitable progression compromising post-implantation stability. Marginal bone resorption usually begins in the cortical bone and progresses toward the apex (Wang et al., 2020). In addition, progressive bone loss is regarded as the first step of peri-implantitis (Galindo-Moreno et al., 2015). Maximum principal stress consists of tensile stress and compressive stress. Previous studies revealed that tensile stress promotes bone deposition while compressive stress promotes bone resorption (Zhong et al., 2013). A satisfactory design should have the ability to effectively disperse stress to avoid excessive stress concentration. Von Mises stress is commonly used in 3D-FEA studies to summarize the overall stress condition and the distribution of compressive stress and tensile stress can be analyzed by a stress distribution map. The maximum force and distribution range map must be taken into consideration to comprehensively determine the ideal design form of Yang’s implant. The ideal design can effectively diminish stress concentration in supporting bone and realize stable implant restoration when the RBH is seriously insufficient. The results showed that the planar style had outstanding advantages. When vertical force was loaded, the maximum von Mises stress in this group could be controlled stably below 40MPa, even when the RBH was only 1 mm left. Oblique forces, which are quite common during normal mastication, cause more stress than axial forces (Tepper et al., 2002). Nevertheless, the PS group could still effectively control the maximum stress value when the RBH was 3 mm. From the map of stress distribution, the planar form had the advantage of dispersing the force concentrated around the hole compared to the liner and triangular groups. The maximum stress induced by the lateral force increases significantly as RBH further decreases. The 3D-FEA established in this paper simulated that the remaining bone volume was only 1 mm, which is an extreme circumstance of severe RBH deficiency in clinical practice. At this time, only the maxillary sinus floor composed of a thin layer of hard compact bone remains (Taschieri et al., 2015). The bone marrow space and blood supply are relatively poor in dense tissue. Blood vessels, capable of transporting oxygen and nutrients, are crucial for bone regeneration (Filipowska et al., 2017). So, in this case, the fresh bone powder implanted by lateral sinus floor elevation would hardly survive. We expect that Yang’s implant with the planar form of wings could be applied in such extreme bone conditions. However, the maximum stress was beyond 200 MPa if a 45° force was loaded. It is a useful method to adjust the inclination of the cusps to eliminate the harmful effects of lateral stress during mastication. A previous study showed when remaining tooth tissue was weak, the stress concentration could be reduced by adjusting the inclination of the tooth cusp after post-core crown restoration, thus reducing the rate of root fracture (Liu et al., 2014). Accordingly, we adjusted the direction of the lateral force, and the different degree of the force simulated cusp slope at different angles of the implant crown. The total stress in the PS group decreased as the force angle reduced. When the force angle was 20°, the maximum stress could be controlled below 150 MPa. Meanwhile, the compressive stress was mainly concentrated on the edge of the hole on the surface of the cortical bone opposite to the direction of the force. The maximum compressive stress shifted from the outer edge to the inner surface of the hole with the increase of the force angle (30°/45°), which could pose a potential risk of bone fracture or mucosa separation.

In summary, Yang’s implant may be a good choice when a severe bone deficiency occurs in the maxillary posterior region. In clinical practice, the planar design form is suggested, which is more conducive to providing stable support in even extreme bone deficiency situations (e.g., RBH is only 1 mm). In such circumstances, it is necessary to properly adjust the cusp inclination of the implant crown to reduce the influence of harmful lateral force. The results of the 3D-FEA are the cornerstone of the large-scale clinical application of Yang’s implants. In the future, the results of this study need to be compared with the accumulated clinical results.

## 5 Conclusion

In our finite element study, two conclusions can be drawn.

1) The customized Yang’s implant can be a less traumatic and invasive method suitable for the implant restoration of patients with severe atrophic maxillary posterior regions. When the RBH in the maxillary posterior region is less than 4 mm, the stress distribution of the short implant with properly designed wings can meet the clinical requirements.
2) Compared to other styles, planar form wings can better disperse the stress and maintain the stability of the implant. By adjusting the cusp slope to reduce the influence of lateral force, the customized short implant with a planar wing fixture can be used safely even if the residual bone height is only 1 mm.

## Data Availability

The raw data supporting the conclusion of this article will be made available by the authors, without undue reservation.
